# Anabolic-androgenic steroid use disorder: case for recognition as a substance use disorder with specific diagnostic criteria

**DOI:** 10.1192/bjp.2025.73

**Published:** 2026-01

**Authors:** Morgan Scarth, Ingrid Amalia Havnes, Astrid Bjørnebekk

**Affiliations:** Anabolic Androgenic Steroid Research Group, Section for Clinical Addiction Research, Division of Mental Health and Addiction, Oslo University Hospital, Oslo, Norway; Division of Mental Health and Addiction, Oslo University Hospital, Oslo, Norway; Institute of Clinical Medicine, University of Oslo, Oslo, Norway

**Keywords:** Diagnosis and classification, substance use disorders, anabolic-androgenic steroids, dependence, performance-enhancing drugs

## Abstract

Approximately one in three people who use anabolic-androgenic steroids (AASs) develop dependence, characterised by both psychiatric and somatic symptoms. Despite this, AAS use disorder (AASUD) is not distinctly recognised in the latest versions of either the ICD or DSM, impeding both clinical care and research progress. It is clear that AASUD shares many features and correlates with substance use disorders (SUDs) that have specific diagnostic criteria in these classification systems, such as stimulants or opioids. We aim to outline the overlap between AASUD and more ‘typical’ SUDs as well as highlight the specific concerns related to AASUD that warrant recognition and distinct diagnostic criteria.

Anabolic-androgenic steroids (AASs) consist of testosterone and synthetic derivatives, which are most commonly used to gain muscle and improve strength and performance. Professional athletes began using AASs to gain a competitive advantage for the 1954 Olympics, and these substances became more popular in subsequent decades, eventually spreading beyond professional athletes in the 1980s.^1,2^ While it is difficult to accurately estimate the prevalence of AAS use because of variations in legal status and cultural norms, a 2014 meta-analysis estimated that the global lifetime prevalence rate is 3.3%, with a higher rate for males (6.4%) compared to females (1.6%).^3^ However, AAS use is more common among certain populations, with 13.4% of athletes and 18.4% of recreational sportspeople reporting lifetime use.^3^ The prevalence among bodybuilders appears even higher, with 36.3% of bodybuilders in Iran and 20.6% in Brazil reporting AAS use.^4,5^ While the majority of people using AASs are male, recent estimates suggest that 16.8% of female bodybuilders and 4.4% of female athletes/recreational gym users have used AASs.^6^

AASs differ from typical psychoactive drugs, as they do not produce a direct intoxication effect and therefore their use is not driven by hedonistic motivations. Rather, they are typically used as a component of a structured diet and exercise plan to achieve a long-term appearance or performance goal. However, there is significant evidence to suggest that AASs have addictive potential. Despite this, AAS use disorder (AASUD) lacks a distinct diagnostic category, posing a challenge to recognising and treating this disorder.

## What is anabolic-androgenic steroid dependence?

The first case report of a patient believed to meet diagnostic criteria for substance dependence based on AAS use was published in 1989, with the patient demonstrating symptoms of tolerance, withdrawal and using AASs to relieve withdrawal symptoms, as well as continued use despite negative consequences.^7^ Research over the last several decades provides strong evidence that AAS use can lead to symptoms of both physical and psychological dependence. For example, AAS withdrawal symptoms result from low testosterone levels (hypogonadism) and may include depression, insomnia and fatigue, as well as headaches, palpitations and myalgia.^8^ Approximately one-third of people who use AASs develop a dependence, a rate higher than that of prescription opioids (16.7%) and similar to cannabis, although the prevalence of opioid and cannabis use remains higher than AAS use.^9–11^ In addition, researchers have proposed several different models of AAS dependence, which include pathways comprising androgenic, anabolic and hedonic effects.^12,13^ Recent work has further specified that AAS dependence is a unique, multidimensional construct encompassing effectiveness, withdrawal symptoms, physical effects, psychological effects and social effects.^14^ Despite this, no internationally recognised medical or psychiatric classification system includes specific diagnostic criteria for AAS dependence.

## Current classification

The term ‘AAS dependence’ is frequently used in research, as most studies related to problematic AAS use are based on adapted diagnostic criteria for *substance dependence* from the DSM-IV.^11,15^ The DSM-IV includes criteria for substance dependence and misuse as distinct categories. However, the term *dependence* can be misleading as it is often understood as the normal physiological adaptations that occur when someone consumes a substance over a period of time, including medications prescribed or recommended by a physician.^16^ Consequently, when the DSM-V was published in 2013, the term *substance use disorder* (SUD) was introduced to capture both the physiological symptoms of substance dependence and the loss of control and compulsive use.^17^ However, AASs would fall under the diagnostic category *other (or unknown) substance use disorder*, as there is no specific diagnostic criteria for AASUD in the DSM. The remainder of this paper will refer to findings based on the DSM-IV criteria using the term *AAS dependence* and *AASUD* for findings based on the DSM-V criteria. AASUD will also be used in the dimensional approach to understanding AAS addiction.

Similarly, the ICD-10 includes codes F10–19 as *mental and behavioural disorders due to psychoactive substance use*, and includes specifiers of *harmful use* and *dependence*, among others.^18^ However, AASs and hormones are not included in this category, but are mentioned under *F55 Abuse of non-dependence-producing substances*, along with laxatives and vitamins.^18^ The ICD-11 includes an update to the language and coding system, introducing the category *disorders due to substance use or addictive behaviors*, with various disorders coded as 6C4X.Y, where X is a specific substance and Y specifies the disorder (harmful episode of use, harmful pattern of use, dependence, intoxication or withdrawal). There are 14 specific substance classes, and it could be assumed that AASs and hormones fall under either 6C4E, *disorders due to use of other specified psychoactive substance, including medications*, or 6C4H, *disorders due to use of non-psychoactive substances.*

However, the accompanying *clinical descriptions and diagnostic requirements for ICD-11 mental, behavioral, and neurodevelopmental disorders* (CDDR) asserts that AASs do not belong within code 6C4H, stating ‘Disorders due to use of non-psychoactive substances do not include disorders related to psychoactive substances such as anabolic steroids. These should be classified under 6C4E Disorders due to use of other specified psychoactive substance, including medications’.^19^ Nonetheless, on the following page, it is stated that ‘Non-psychoactive substance use may occur in the context of other mental disorders (e.g. use of laxatives in anorexia nervosa to reduce body weight, use of anabolic steroids in body dysmorphic disorder to increase muscle mass)’, creating ambiguity around whether AASs should be considered psychoactive or not. If we assume that AASs can be included under 6C4E, as is clearly stated (page 448), there are still significant challenges to identifying AAS-specific symptoms using the CDDR, as there are no specific symptoms provided in the CDDR for substances in this class.

The inclusion of diagnostic criteria specific to AASUD is important for several reasons, including recognition of AASUD as a disorder comprising both physical and psychiatric symptoms. Furthermore, specific diagnostic criteria will increase awareness and perceived validity of AASUD in both clinical and research settings, making this disorder easier to recognise, diagnose and study, and ultimately lead to improved knowledge and treatment options. In addition, research on AASUD and dependence largely focuses on men. However, evidence suggests that while women do experience symptoms of AAS dependence, they may exhibit lower levels of tolerance and withdrawal compared to men, indicating a need for gender-specific considerations in diagnostic criteria.^20^ Including AAS-specific symptoms and gender-specific criteria would be highly beneficial for both clinicians and patients seeking treatment for AAS-related symptoms, which will be explored in greater detail below. Notably, research on AAS dependence in women is scarce, and no studies have measured craving. Thus, additional research is needed to better understand physical and psychological symptoms of AASUD among women.

## Comparison of anabolic-androgenic steroid use disorder and substance use disorder

Table 1 presents a summary of comparisons between AASUD and SUDs, including sociodemographic and neuropsychiatric characteristics, and withdrawal symptoms. It is clear that there is significant overlap between AASUD and addiction to psychoactive substances and behaviours. While the field of AASUD is relatively small compared with more common SUDs, including alcohol and opiates, research over the last few decades indicates several similarities with regard to neuropsychological, psychiatric and psychosocial characteristics among people with SUD and people with AAS dependence. Importantly, there are also some key distinctions between these groups, highlighting the importance of substance-specific criteria.

**Table 1:** Comparison of anabolic-androgenic steroid use disorder and other substance use disorders across sociodemographic factors, neuropsychiatric characteristics and withdrawal symptoms

Variables	Anabolic steroid dependence/use disorder	Substance use disorder
Age of initiation	Earlier start increases the risk for dependence	Increased risk for dependence with young initiation age, for example drinking alcohol before 15
Demographics	Highest incidence among male bodybuilders, inmates in prison, people in substance abuse treatment and ‘recreational athletes’	Substance use disorders most common among men, increasing incidence among women especially with prescribed opioids
Common psychiatric comorbidities	Body image disorder, depression, anxiety, antisocial personality disorder, perfectionism, ADHD	Depression, anxiety, antisocial personality disorder, ADHD
Cognitive function	Reduced executive function (including ability to initiate, control and monitor goal-directed behaviour), reduced recognition of fear, reduced capacity to take others’ perspective	Reduced executive function, reduced mental flexibility and working memory, reduced recognition of negative emotions, altered empathy
Brain effects	Reduced volume prefrontal cortex (important for processing information, planning and making decisions), thinner cortex in areas of inhibitory control, changes in the reward system	Reduced volume prefrontal cortex, thinner cortex in areas of inhibitory control, changes in the reward system
Acute impact	Mild psychoactive effect: increased sense of well-being, increased self-confidence, increased energy	Acute impact on the brain’s reward system, euphoria, well-being
Withdrawal/abstinence		
When	Time for AAS/testosterone to be excreted from the body can vary from several weeks up to months based on half-life	From hours (heroin) to days (tranquilisers)
Systems involved	Endocrine Neuropsychiatric Autonomic nervous system	Neuropsychiatric Autonomic nervous system
Symptoms	Hypogonadism disturbance in the hormonal system. Symptoms of low testosterone levels: severe fatigue, reduced sexual desire (libido), erectile dysfunction Neuropsychiatric depression with/without suicidal thoughts, anxiety, sleep problems, ‘cravings’, body image disturbance – psychological reaction to less muscle volume Autonomic nervous system headache, nausea, extra heartbeat, muscle tremors	Substance-dependent, but general: restlessness, difficulty concentrating, intense urge to take the drug, lethargy, difficulty experiencing pleasure. Specific symptoms for each drug, such as opioids: vomiting, fever, sweating, body aches, tremors, diarrhoea

ADHD, attention-deficit hyperactivity disorder; AAS, anabolic-androgenic steroid.

Several characteristics, including male gender, early life adversity, low levels of education and early exposure to substances constitute risk factors for SUDs as well as AAS dependence.^11,21^ At the same time, there are distinct characteristics and exposures that may partially explain why individuals develop different types of addictions or SUDs.

### Cognitive and neurobiological factors

Addiction has been conceptualised as a chronic relapsing disorder comprising three stages: *binge/intoxication*, *withdrawal/negative affect* and *preoccupation/anticipation*, which correspond to distinct brain structures and circuits.^22^ During the *binge/intoxication stage*, the positive and rewarding effects of drugs are experienced. While AASs do not induce a typical intoxication syndrome, people using AASs frequently report feelings of mild euphoria and invincibility.^23,24^ Similarly, animal models suggest that reward circuitry implicated in addiction is also affected by AAS use.^25,26^ AASs also differ from most psychoactive drugs in that they do not usually cause acute impairments in coordination, perception or cognition, and therefore do not require the same level of regulation with respect to tasks such as driving, compared to substances like alcohol or opioids. This may partially explain why individuals evaluated for AAS dependence or AASUD infrequently report failing to fulfil social or occupational obligations because of use.^27–29^

The *withdrawal/negative affect* stage begins when substance use is ceased or reduced, and people with dependence will experience symptoms of withdrawal, including increased irritability and anxiety. Withdrawal symptoms can vary depending on the substance, with withdrawal from psychoactive substances resulting from central nervous system adaptations, leading to both neuropsychiatric and physical symptoms. For example, opioid withdrawal may include fever, nausea and vomiting, excessive sweating and yawning, as well as increased anxiety, irritability and insomnia.^30^ People using AASs often report withdrawal symptoms; however, these symptoms are largely driven by the endocrine system, particularly the hypothalamic–pituitary–gonadal (HPG) axis, in addition to neuropsychiatric effects related to the autonomic nervous system.

The acute phase of AAS withdrawal can include sympathetic activation with symptoms of headache, palpitations and nausea.^31^ In addition, AASs are distinct from non-hormonal drugs in their impact on endogenous hormone production, which can lead to enduring withdrawal symptoms. The administration of supraphysiological doses of androgens suppresses the release of key reproductive hormones, leading to reduced endogenous testosterone production caused by a negative feedback mechanism within the HPG axis, known as hypogonadism. This negative feedback loop is intensified by high levels of oestrogens resulting from aromatisation of exogenous AASs. These changes to the HPG axis contribute to symptoms of testosterone deficiency when AAS use has ceased, including fatigue, erectile dysfunction, depression and low libido.^8^ While hypogonadism is also highly prevalent among individuals using opioids, it is not directly related to the development of opioid use disorder, highlighting the unique role of hormonal dysregulation in AAS withdrawal and its distinct impact on the motivation to continue using. Notably, in preliminary research we have seen that women report symptoms of withdrawal less often than men, which may be explained by lower endogenous testosterone levels in women and should be taken into consideration in clinical evaluations.^20^

Finally, the *preoccupation/anticipation* stage is characterised by craving a substance and involves executive functions, including impulse control, which are mediated by structures such as the prefrontal cortex. Deficits in executive function and attention are highly prevalent among people with SUD, leading to challenges such as poor impulse inhibition, emotion regulation and decision-making.^32–34^ These functions are critical to inhibiting craving, which was added to the DSM-V as a SUD symptom. To our knowledge, two studies have used this criteria to evaluate AASUD, finding that 27.9% and 42.7%, respectively, of people reporting AAS use experienced craving, which was the most commonly reported AASUD symptom in both studies.^27,28^ In addition, a recent study evaluating an AAS craving scale identified underlying aspects of craving, including expectation, environment and mood.^14^ These findings may contribute to a deeper understanding of this symptom in the context of AAS use, which has its own distinct environment and use patterns.

Interestingly, people with AAS dependence often exhibit similar patterns of executive dysfunction relative to other SUDs, including compromised impulse inhibition, working memory and emotional control.^35,36^ In addition, brain imaging studies have identified a thinner cortex in prefrontal regions associated with inhibitory control,^37^ and signs of accelerated brain ageing in frontal regions among people with AAS dependence relative to controls,^38^ a pattern also often observed in other drug dependencies.^39,40^

### Psychiatric comorbidities

SUDs are frequently comorbid with other psychiatric conditions. It is difficult to determine the direction of this relationship, as early mental illness may contribute to problematic substance use, or substance use may contribute to the development of mental illness. Furthermore, it may also be the case that there are shared underlying vulnerabilities for multiple disorders. Regardless, it is clear that there is a higher prevalence of psychiatric disorders, including depression, anxiety, post-traumatic stress disorder, attention-deficit hyperactivity disorder (ADHD) and several personality disorders, including antisocial and borderline, among people with SUD compared to the general population.^41,42^

Similarly, AAS use and dependence have been associated with a number of psychiatric conditions, many of them overlapping with those observed in other SUD populations. These include ADHD, anxiety, depression and several personality disorders, including antisocial, paranoid and borderline (now called *emotionally unstable personality disorder* in the ICD-11).^36,43–46^ In addition, it is important to highlight the role of body dysmorphia, in particular muscle dysmorphia, among people using AASs. People with these body image concerns are more likely to use AASs and other supplements or behaviours that put their health at risk in the pursuit of improving musculature and appearance.^29,47,48^ Furthermore, social physique anxiety and fears of losing muscle mass may prolong AAS use and increase the severity of AAS dependence.^49^

### Motivation and social context

The motivation to use AASs differs from other substances, as AASs are most often used to achieve a long-term physique or performance goal. AASs are usually one component of a structured regimen involving rigorous training and adherence to a strict diet. This goal-driven mindset contrasts with classical psychoactive drugs, where use is often driven by immediate gratification, either seeking euphoria, or relief of pain, stress or emotional discomfort.^50^ This distinction in motivational forces is likely also one reason why people who use AASs may not identify with SUD patients.^51^ However, these groups likely share underlying vulnerabilities, such as low self-esteem and cognitive, genetic and environmental factors, as described previously.

Understanding the distinction in motivational forces is critical, as these variations may inform barriers to seeking treatment. The long-term focus and structured, intentional use of AASs may make it more difficult for individuals using these substances to recognise when the negative health effects overshadow the positive ones. Many people who use AASs are striving to achieve an ‘ideal’ physique that is perceived as optimal within certain communities, making it challenging to cease use and seek treatment, even when the adverse effects become concerning to the individual.

The social and physical environment in which AASs are used differ substantially from those associated with psychoactive drugs like alcohol or opioids. While social conformity can play a role in all types of addictions, it is important for health professionals to be aware of the specific context in which AASs are used. Use of AASs can be closely tied to subcultures such as the gym culture and/or bodybuilding communities. Within these environments, AAS use might be normalised as a legitimate part of a typical training regime aimed at enhancing muscle mass and physical appearance.^52^ In addition, AASs are often used in conjunction with other appearance and performance-enhancing drugs, including stimulants, insulin, growth hormones and drugs taken to reduce fat, such as clenbuterol.

AAS use may also be driven by societal pressure to adhere to traditional ideals of masculinity, or to fend off ageing that men may attribute to declining testosterone levels.^53,54^ These social contexts and environments can distinguish AAS users from those using substances like 3,4-methylenedioxymethamphetamine (MDMA) or heroin and may contribute to their reluctance to identify with or seek treatment in facilities primarily designed for other SUDs.

However, while not the primary population using AASs, a high prevalence of AAS use has been reported among people in SUD treatment (28%), which may be explained by specific motivations, including a desire to regain weight and muscle mass following prolonged use of opioids or stimulants.^55^ Furthermore, several shared risk factors, including adverse childhood experiences, ADHD and certain personality disorders, may explain the significant overlap between AAS use and other substance use.

### Diagnostic considerations

There is strong evidence that AAS use can lead to both physical and psychological dependence, underscoring the need for a specific diagnostic category for AASUD. While AASUD shares many cognitive, neurobiological and psychiatric features with other SUDs, there are unique challenges that warrant specific clinical attention. Unlike typical psychoactive substances, AASs directly affect the endocrine system, disrupting reproductive hormones and often resulting in reduced or absent testosterone production. Establishing specific diagnostic criteria for AASUD will enhance healthcare professionals’ awareness and understanding of the disorder, as well as the unique challenges faced by patients. This is a particularly critical point, as people using AASs often perceive doctors to lack competence in AAS knowledge, and subsequently prefer self-treatment methods along with advice from peers.^56,57^

**Fig. 1:**
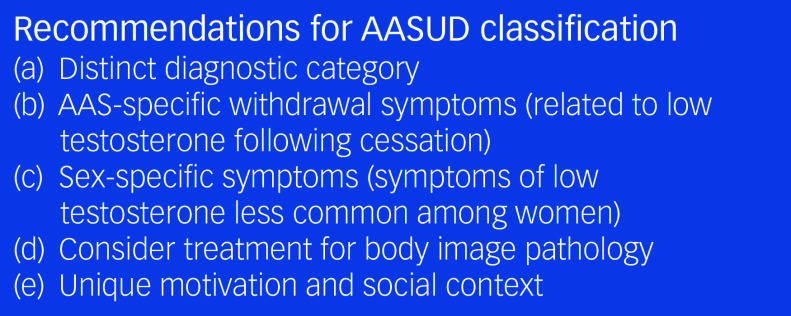
Recommendations for AASUD diagnostic classification.

Figure 1 summarises important points for diagnostic criteria for AASUD. Both the ICD and DSM should include information about AASUD, including its association with body image pathologies and the unique social and environmental contexts in which AASs are used. Descriptions of withdrawal symptoms should highlight the impact of AASs on the HPG axis and reproductive hormones. Gender differences should also be mentioned, particularly that women may not experience withdrawal symptoms to the same extent as men, but may face additional stigma and shame associated with AAS use.

Finally, the relevance of failing to fulfil social or occupational obligations because of use should be carefully considered for AASUD, as AASs do not inhibit people using them to the same extent as other types of drugs, and this symptom often has low prevalence among people reporting AAS use. With these considerations, healthcare providers will be better equipped to recognise, diagnose and treat AASUD, with significant benefits for patients.

## Data Availability

Data availability is not applicable to this article as no new data were created or analysed in this study.
